# The Wisdom of Honeybee Defenses Against Environmental Stresses

**DOI:** 10.3389/fmicb.2018.00722

**Published:** 2018-05-01

**Authors:** Guilin Li, Hang Zhao, Zhenguo Liu, Hongfang Wang, Baohua Xu, Xingqi Guo

**Affiliations:** ^1^State Key Laboratory of Crop Biology, College of Life Sciences, Shandong Agricultural University, Tai'an, China; ^2^College of Animal Science and Technology, Shandong Agricultural University, Tai'an, China

**Keywords:** honeybee, environmental stress, abiotic stress, biotic stress, defense mechanism

## Abstract

As one of the predominant pollinator, honeybees provide important ecosystem service to crops and wild plants, and generate great economic benefit for humans. Unfortunately, there is clear evidence of recent catastrophic honeybee colony failure in some areas, resulting in markedly negative environmental and economic effects. It has been demonstrated that various environmental stresses, including both abiotic and biotic stresses, functioning singly or synergistically, are the potential drivers of colony collapse. Honeybees can use many defense mechanisms to decrease the damage from environmental stress to some extent. Here, we synthesize and summarize recent advances regarding the effects of environmental stress on honeybees and the wisdom of honeybees to respond to external environmental stress. Furthermore, we provide possible future research directions about the response of honeybees to various form of stressors.

## Introduction

Honeybees (Hymenoptera: Apidae), highly eusocial insects, first emerged ~120~130 million years ago, coinciding with the appearance of early angiosperms (Engel, 2001). With continued study, it is discovered that honeybees are essential to the agricultural economy because of their efficient pollination of many agricultural crops worldwide. Estimates suggest that crop yields will decrease by more than 90% without honeybee pollination (Klein et al., 2007). Honeybees are also significant for many wild plant communities (Potts et al., 2010). Some plants even have unique reproductive structures that, through the process of evolution, only allow pollination by honeybees. Moreover, honeybees can make important contributions to human life through their ability to produce honey, propolis, bee venom, bee wax, and royal jelly.

**Figure 1:**
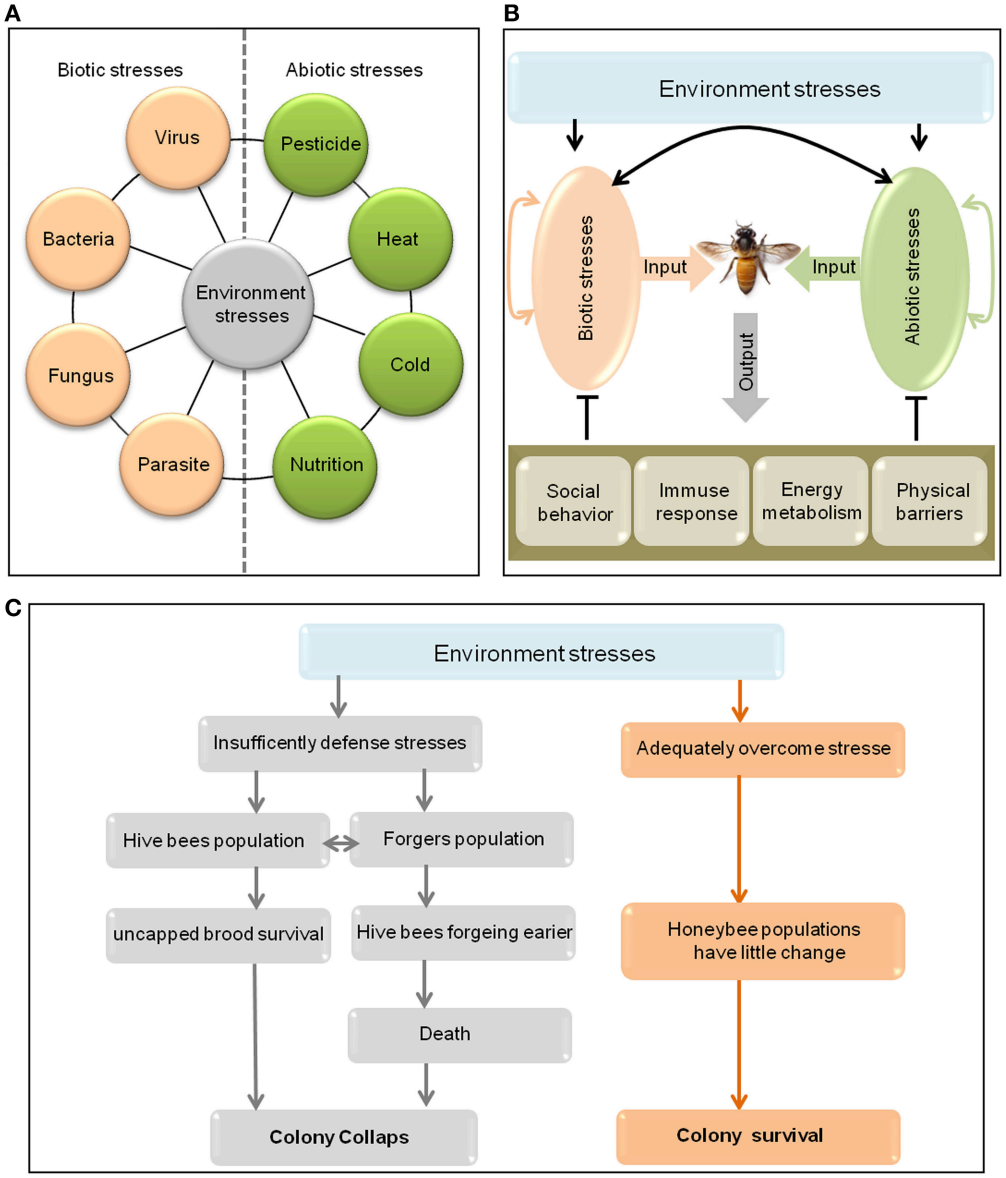
Environmental stresses on honeybees and the possible responses of honeybees to them. **(A)** A summary of some environmental stresses on honeybees. **(B)** Though different stress agents can interact with each other to affect the health of honeybees, the honeybees will defend against stress by using social behavior, immune response, physical barriers, and changing in energy metabolism. **(C)** Environmental stresses may lead to honeybee colony collapse or survival. On the one hand, if honeybees cannot bear the stress, both the hive bee and forager populations will decrease. The decrease in the hive bee population has negative effects on uncapped brood survival and the forager population. The decrease in the forager population triggers the early maturation of hive bees to become foragers, which have a high death rate, will also affect the hive bee population, and eventually drives the rapid depopulation of stressed honeybee colonies (Park et al., 2015). On the other hand, if the colony can adequately overcome the stresses, the colony population may change only slightly, and the colony is likely to survive.

Although the important roles of honeybee in agricultural productivity, wild plant communities and human livelihoods, there is clear evidence of marked regional population decreases in honeybee populations due to colony failure in recent years (Potts et al., 2010; Kulhanek et al., 2017). In particular, from the winter of 2006 to the spring of 2007, adult bees suddenly underwent mass disappearances in outwardly healthy colonies within ~2–4 weeks in parts of Asia, Europe and the United States, leading to alarming levels of colony failure, termed colony collapse disorder (CCD) (Vanengelsdorp et al., 2009). Such catastrophic colony losses may seriously influence wild plant diversity, terrestrial ecosystem stability, crop production, global food supply, and human welfare. A general consensus now exists that CCD is a product of environmental stress, including multiple forms of biotic and abiotic stress (Figure 1A) that function singly or synergistically (Potts et al., 2010; Ratnieks and Carreck, 2010; Vanbergen, 2013; Kerr et al., 2015; Perry et al., 2015). Though high levels of environmental stress will lead to honeybee brought to their knees to it, honeybees can cope with low levels of environmental stress through their “wisdom,” such as decreasing the natural mortality of foragers, reducing the forager recruitment level and increasing queen's laying rate (Ratnieks and Carreck, 2010; Evans and Schwarz, 2011; Vanbergen, 2013; Booton et al., 2017). Moreover, we previously found that many genes and signaling pathways can also be employed by *Apis cerana cerana* for defense against environmental stresses (Yu et al., 2011; Meng et al., 2014; Yao et al., 2014; Li et al., 2016a,b; Zhang J. et al., 2016; Zhang Y. Y. et al., 2016; Zhao et al., 2018).

## Biotic stress in honeybees

Biotic stresses in honeybee mainly include fungi, bacteria, virus and parasites (Figure 1A), which can infect different developmental stages of honeybees, resulting in various diseases and pathogenic characteristics (Table 1; Schmidhempel, 1998; Randolt et al., 2008; Albert et al., 2011; Evans and Schwarz, 2011; Gatschenberger et al., 2013; Collison et al., 2016). The honeybee genome lacks many typical innate immunity genes that enable other insects to cope with biotic stress (Schmid-Hempel, 2005; Evans et al., 2006; Lemaitre and Hoffmann, 2007; Rolff et al., 2009). However, this does not mean that honeybees are defenseless against pathogens and parasites. Instead, they have evolved a suite of efficient defensive measures to fight the invasion of various pathogens and parasites, including social behavior (Box 1), energy metabolism, innate immune response (Glossary), and physical barriers such as gut epithelium and cuticle (Figure 1B; Cremer et al., 2007; Randolt et al., 2008). Not only that, honeybees can use many other mechanisms to respond to biotic stress, and the defense mechanisms of honeybee against multiple stress agents exhibit different characteristics.

**Table 1:** A summary of some parasites and pathogens of the honeybees.

**Classification**	**Pathogenic characteristics**	**Species**	**Cause of disease**	**Susceptible developmental stages**	**References**
Virus	Existence with no obvious symptoms until activated under certain environmental conditions, resulting in strong pathogenicity	Black queen cell virus	Black queen cell disease	Larvae and pupae	Ellis and Munn, 2005; Zhang et al., 2012
		Deformed wing virus	Deformed wing disease	Various life stages	Bailey and Ball, 1991
		Israeli acute paralysis virus	Israeli acute paralysis disease	Various life stages	de Miranda et al., 2010
		Sac brood virus	Sacbrood bee disease	Various life stages	Bailey and Fernando, 2010; Park et al., 2016
		Chronic bee paralysis virus	Chronic bee paralysis disease	Adults	Bailey and Woods, 1974
		Kashmir bee virus	Kashmir bee disease	Various life stages	Ellis and Munn, 2005
		Acute bee paralysis virus	Acute bee paralysis disease	Various life stages	Bailey, 1981
		Tobacco ringspot virus	Unclear	At least adult worker bees	Li et al., 2014
Bacteria	Long infection time, fast propagation speed and serious harm	*Spiroplasma apis*	Spiroplasmosis	Adults	Mouches et al., 1983
		*Spiroplasma melliferum*	Spiroplasmosis	Adults	Clark et al., 1985
		*Paenibacillus larvae*	American foulbrood disease	Larvae	Morse and Nowogrodzki, 1978
		*Melissococcus plutonius*	European foulbrood disease	Larvae	Morse and Nowogrodzki, 1978
Fungi	Frequent association with rain, humidity and unstable temperature	*Ascosphaera apis*	Chalkbrood disease	Larvae	Mehr et al., 1978
		*Aspergillus flavus*	Aspergillus flavus disease	Various life stages	Chen, 2013a
Parasite	Ingestion of nutrients in honeybees	*Nosema ceranae*	Microsporidiosis	Adults	Sak et al., 2004
		*Nosema apis*	Microsporidiosis	Adults	Sak et al., 2004
		*Tropilaelaps*	Acariasis of bees	Larvae	Chen, 2013b
		*Varroa destructor*	Acariasis of bees	Larvae, pupae and adults	Martin, 2001

Box 1 **Social Behavior**

Honeybees can use many social behaviors to address microbial infection. For example, the honey produced by honeybees contains specific ingredients with antimicrobial activity, including methylglyoxal, sugar, H_2_O_2_, and defensin1, which can be used by honeybee to defend against pathogens and parasites. Honeybees can also collect certain compounds that possess potential antimicrobial properties, such as resins, plant pollens, and complex plant secretions. These compounds can be further used by honeybee to produce propolis to coat colony interior and thereby decrease pathogen loads in the colony (Cremer et al., 2007; Simone et al., 2009; Wang et al., 2015; Wang K. et al., 2016). Moreover, honeybees can perform allogrooming and hygienic behavior, change worker-worker interactions, switch from in-hive tasks to foraging, and remove ectoparasites and diseased brood to reduce the transmission of pathogens and parasites between individuals in the hive (Rueppell et al., 2010).

### Fungi

The main fungi that threaten honeybee colonies include *Ascosphaera apis* and *Aspergillus flavus*, which trigger chalkbrood disease and aspergillosis, respectively (Table 1). Infection with these fungi often accompany rain, humidity and unstable temperature (Mehr et al., 1978; Chen, 2013a). Foundation wax contaminated with *A. apis* spores is a likely source of chalkbrood disease in honeybee colonies (Flores et al., 2005). The lethality of *A. apis* is dose dependent and can be exacerbated by environmental humidity and temperature (Aronstein and Murray, 2010).

Transcriptome sequencing analysis reveals many pathways involved in cellular immune, humoral immune-related, MAPK signaling, Toll-like receptor signaling, and part of the nuclear factor-κB (NF-κB) signaling are upregulated in the gut of *Apis mellifera ligustica* infected with *A. apis* (Chen et al., 2017), indicating that honeybees can employ these signaling pathways to cope with *A. apis* infection. In addition, 3-acyl dihydroflavonols (a component of poplar resins) and the extracts of many plants, such as *Allium sativum, Piper betle, Syzygium aromaticum, Amomum krervanh, Piper sarmentosum, Cinnamomum* sp., and *Piper ribesioides* have been proved to exhibit inhibitory effect on *A. apis* by utilizing antimicrobial activity assays (Chaimanee et al., 2017; Wilson et al., 2017). These substances can be used by beekeepers to prevent chalkbrood disease in the future. It is worth mentioning that the larval mortality caused by *A. apis* is increased by cooling the larvae for 24 h after inoculation (Vojvodic et al., 2011), suggesting that cold may facilitate *A. apis* infection. Of course, further research is needed to confirm the interaction between *A. apis* infection and cold stress.

Regarding *A. flavus*, an omega-class glutathione S-transferase has been found to participate in the response to *A. flavus* infection in *Apis cerana cerana* (Zhang Y. Y. et al., 2016). The acetone, hexane and petroleum ether extracts from thyme and santonica plants at 1,000 parts per million completely repress the growth of *A. flavus* (Ali, 2007), and thus may be used by beekeepers to control *A. flavus* infection in honeybee colonies.

### Bacteria

The main bacterial pathogens that infect honeybees are *Paenibacillus larvae, Melissococcus pluton, Spiroplasma melliferum, Pseudomonas*, and *Salmonella paratyphi A*, which can cause American foulbrood, European foulbrood, honeybee spiroplasmosis, septicemia, and paratyphoid disease, respectively (Table 1; Morse and Nowogrodzki, 1978; Mouches et al., 1983; Clark et al., 1985; Evans and Schwarz, 2011). These diseases have long infection times and fast propagation speed, and cause serious degree of harm that severely influences the growth and development of honeybees (Evans and Schwarz, 2011).

For the first 48 h following eclosion, honeybees are susceptible to infection by *P. larvae*. However, honeybees become progressively more resistant to this bacterial pathogen with age (Brødsgaard et al., 1998). That may be caused by the age-dependent development of the gut epithelium, which is a barrier for *P. larvae*, and too small synthesis of antimicrobial compounds to produce adequate amounts of antimicrobial peptides in first instar larvae (Yue et al., 2008; Gatschenberger et al., 2013). Based on mass spectrometry-based proteomics analysis, the levels of chaperones, immunity proteins, and certain metabolic proteins are found to be upregulated in the hemolymph of 5-day-old healthy larvae compared with their levels in infected honeybee larvae. These findings reveal that honeybee larvae can not only fight *P. larvae* infection directly, by using immune factors, but also indirectly, by employing energy metabolism pathways to sustain the effort (Figure 1B; Chan and Foster, 2008; Chan et al., 2009). Interestingly, a result from immunohistochemical localization technique reveals that heat shock protein 70 (Hsp70) localizes in the cytoplasm and nuclei of hemocytes, midgut cells, and salivary gland cells in honeybee larvae infected with *P. larva* but not in uninfected honeybee larvae (Gregorc and Bowen, 1999), which suggests that Hsp70 may be used as a possible diagnostic criterion for *P. larvae* infection. Many plant extracts, for example *Chromolaena odorata* and longer acyl groups can be employed to defend against *P. larvae* infection (Chaimanee et al., 2017; Wilson et al., 2017).

**Figure 2:**
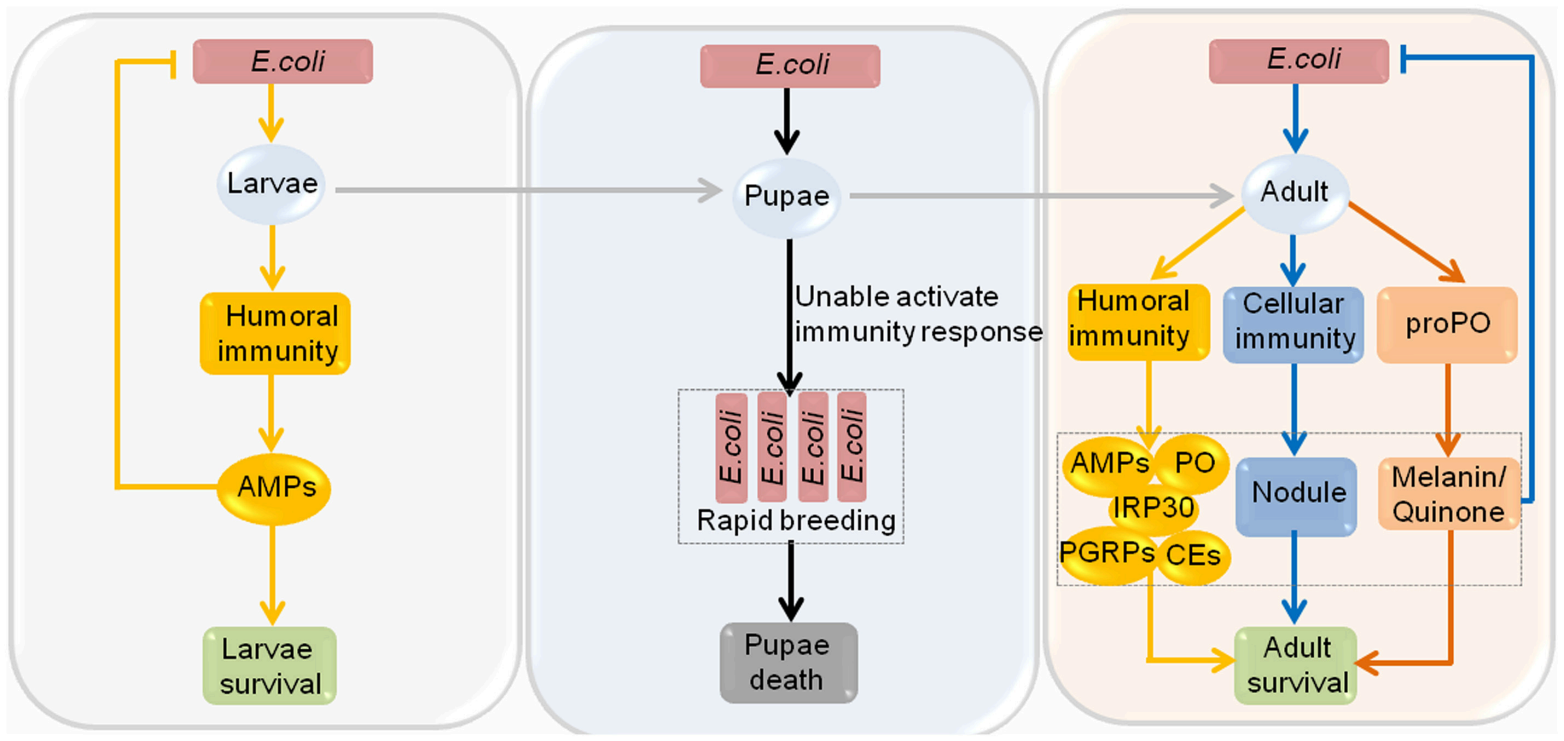
The immunocompetence of honeybees at different developmental stages to *Escherichia coli* infection. The immunocompetence of honeybees to *Escherichia coli* (*E. coli*) infection varies among different development stages. Honeybees go through the larval stage and then the pupal stage before becoming adult. High-dose injection of *E. coli* into honeybee larvae causes a humoral immunity response and then induces the synthesis of antimicrobial peptides (AMPs), including defensin1, abaecin and hymenoptaecin, which can repress the multiplication of *E. coli*, and the larvae can survive. Pupae are completely incapable of activating cellular and humoral immune reactions upon *E. coli* infection, resulting in the rapid proliferation of *E. coli*, ultimately triggering the death of pupae. Moreover, both the humoral and cellular immune responses play important roles when adult honeybees are infected with *E. coli*. Prophenoloxidase is activated, resulting in synthesis of melanin and quinones. In the humoral immune response, AMPs, phenoloxidase (PO), carboxylesterases (CEs), peptidoglycan recognition proteins (PGRPs) and immune responsive protein 30 (IRP30) are detected, and in the cellular immune response, nodules are formed. All of these immune reactions can repress the amplification of *E. coli*, resulting in the survival of adults.

Aside from *P. larvae*, honeybees are also susceptible to certain gram-negative bacteria, such as *Escherichia coli* (*E. coli*). Gene ontology analysis shows that canonical immune response pathways, particularly the Notch signaling pathway and the Toll signaling pathway are specifically altered in response to *E. coli* infection in *Apis mellifera* (Richard et al., 2012). These signaling pathways may play important roles in defending against *E. coli* infection in honeybees. Interestingly, the immunocompetence competence of honeybees to *E. coli* infection varies between different development stages, different seasons, and different castes honeybees. Honeybee larvae and adult can use immune responses to defend against *E. coli* infection. However, pupae have no immune system, thus they cannot cope with the infection of *E. coli* through immune response (Figure 2). Moreover, summer adult honeybees upregulate seven types of immune proteins, namely, defensin1, abaecin, hymenoptaecin, phenoloxidase (PO), carboxylesterases (CEs), peptidoglycan recognition proteins (PGRPs), and immune responsive protein 30 (IRP30), after infection with *E. coli*, while only hymenoptaecin, defensin1 and IRP30 are induced in winter honeybees. It is worth mentioning that winter honeybees have no nodulation reactions as observed in summer honeybees, but they possess an enlarged fat body and many haemocytes, which allow them to kill viable *E. coli* faster and more reliably than summer honeybees. In regard to different castes of honeybees, after infection with *E. coli*, hymenoptaecin, defensin 1, abaecin, PGRPs and lysozyme 2 are detected in drones, while only hymenoptaecin, CEs and IRP30 are found in queens (Randolt et al., 2008; Albert et al., 2011; Gatschenberger et al., 2012, 2013). Workers infected with *E. coli* tend to increase allogrooming behavior, alter their social interactions, and become more aggressive. However, the defense mechanism of honeybee eggs to *E. coli* remains unclear, and should be the focus of future study.

### Viruses

Viruses are widely involved in colony diseases, and are both historically and recently known to be harmful to the physiology, behavior, morphology and learning ability of honeybee (Bailey and Ball, 1991; Chen et al., 2005; Maori et al., 2007; Runckel et al., 2011). More than 20 viruses are reported to infect honeybees (Bailey and Woods, 1974; Bailey, 1981; Ellis and Munn, 2005; Bailey and Fernando, 2010; de Miranda et al., 2010; Zhang et al., 2012; Li et al., 2014). Among these viruses, Deformed wing virus (DWV) infection causes deformed wings, small body size and discoloration in adult honeybees (Bailey and Ball, 1991). Though the viral load of DWV increases slowly before the adult stage of honeybee, and this virus seldom kills pupae, it decreases the total lifespan of honeybee by acting independently or synergistically with *Varroa destructor* (Dainat et al., 2012). If the copy number of DWV reaches a specific threshold, it will restrain the immune system of honeybee, then accelerates greater replication of DWV (Highfield et al., 2009; Dainat et al., 2012; Wu et al., 2017). Feeding larvae in advance with DWV double-stranded RNA (DWV-dsRNA) reduces wing deformity in adults, and feeding adults with DWV-dsRNA reduces the DWV concentration and increases adult longevity (Dainat et al., 2012). The oral administration of 0.5% β-glucan (Glossary) can enhance the tolerance of *Apis mellifera ligustica* to DWV, possibly by increasing phenoloxidase activity and the number of prohemocytes (Mazzei et al., 2016).

Chinese sacbrood virus (CSBV) causes sacbrood disease in *Apis cerana* with low prevalence and pathogenicity (Gong et al., 2016). In-situ hybridization can be used to detect, diagnose, and locate CSBV (Park et al., 2016). Gel-based and liquid chromatography-mass spectrometry-based proteomic strategies have shown that networked groups connected with the cytoskeleton, development, protein metabolism, protein folding, and energy metabolism are clearly changed in honeybee larvae suffering from sacbrood disease. In addition, the antioxidant defenses of honeybee are overwhelmed by CSBV infection. All these changes severely influence the normal development of larvae, and prevent the metamorphosis from larvae to pupae (Aronstein and Murray, 2010; Han et al., 2013). Recent studies demonstrate that RNA interference (RNAi) is a valuable antiviral strategy for sacbrood disease control. For example, a marked reduction in larval mortality is found after bee ingesting of dsRNA-VP1 that against VP1 gene in CSBV (Liu et al., 2010; Zhang J. et al., 2016). Therefore, if possible, RNAi can be considered for use in defense against CSBV in beekeeping.

Israeli acute paralysis virus (IAPV) is thought to be closely related to CCD in the US (Cox-Foster et al., 2007). Hou et al. support this view by showing that when infected with high loads of IAPV, the colonies in an apiary present typical CCD characteristics (Hou et al., 2014). *V. destructor* can act as a vector of IAPV, and the IAPV copy number is positively related to the density of *Varroa* mites (Di Prisco et al., 2011). By using mass spectrometry-based quantitative proteomics analysis and gene expression analysis, it is revealed that various pathways related to mitosis, cell division, energy production and the protein biosynthetic machinery and many fundamental cellular processes connected with ribosomal biogenesis and other cellular functions are affected or disturbed by IAPV infection in *Apis mellifera* (Boncristiani et al., 2013; Michaud et al., 2014). Changes in these pathways and cellular processes may severely impact the lives of honeybees, even causing their death. Honeybees fed with dsRNA-IAPV can successfully silence IAPV, resulting in greatly decreased honeybee mortality triggered by IAPV infection (Maori et al., 2009). Therefore, using dsRNA-IAPV in apiculture may be an effective strategy to protect honeybee hives from IAPV and even from CCD.

Recent studies show that a dose of 10^4^ virus particles of Acute bee paralysis virus (ABPV) per individual causes typical paralysis and trembling signs, and is followed by death within 48 h in adult bees. The ABPV dose of 10^3^ virus particles per individual retards growth, and triggers a change from yellowish-white color to brownish-black followed by the sudden collapse of the infected bee larvae (Randolt et al., 2008; Fedorova et al., 2011). When present on its own, ABPV has a low impact on the survival of whole honeybee colony, while accompanied by infection with *V. destructor*, ABPV is related to high colony mortality (Genersch et al., 2010). Though the innate immune response plays indispensable roles in counteracting bacterial and some viral infections (Trenczek, 1998; Lemaitre and Hoffmann, 2007), a previous study demonstrates that infection with ABPV does not cause cellular or humoral immune responses in young adult worker bees or honeybee larvae. Instead, proteins involved in translation, antioxidant protection, stress response and energy metabolism are upregulated when honeybees are infected with ABPV (Azzami et al., 2012). These proteins may play important roles for honeybees to withstand ABPV infection. A recent study shows that aRNases D3-12 and Dp12F6 exhibit high cleavage activity of ABPV RNA accompanied by low toxicity and without changing the morphology of ABPV particles, resulting in ABPV inactivation and increased survival rate of adult and larval bees that infected with ABPV (Fedorova et al., 2011). This result, together with the above-mentioned considerable difference in the influence of ABPV on adult and larval bees, as well as the lack of acquired immunity, make bees a possible new experimental model for the identification of antiviral agents (Fedorova et al., 2011). If aRNases D3-12 and Dp12F6 can be used to produce novel vaccines that are extensively used in apiculture in the future, the damage caused by ABPV will certainly be significantly decreased.

### Parasites

Some parasites are the carriers of viruses that infect honeybees, and parasite infection leads to physical decline in honeybees (Martin et al., 2012; Wu et al., 2017). *Nosema ceranae, Malpighamoeba mellificae* and mites are the most common honeybee parasites, and they can trigger microsporidiosis, loeschiasis and acarine disease, respectively (Martin, 2001; Sak et al., 2004; Chen, 2013b). Among these parasites, *N. ceranae* was first identified in the Asian honeybee (*Apis cerana*) and then transferred to the Western honeybee (*Apis mellifera*) as a host. It can synergistically interact with DWV in a nutrition- and dose-dependent manner (Zheng et al., 2015), suggesting a connection among DWV, *N. ceranae*, and nutrition. The optimal temperature for *N. ceranae* infection is 25°C, and the preferred temperature for its multiplication is 35°C (Woyciechowski and Czekonska, 2014), which may explain the phenomenon that honeybees infected with *N. ceranae* are inclined to congregate in the warmer part of the hive (Moeller, 1956).

Recent researches demonstrated that *N. ceranae* degenerates honeybee gut epithelial cells, increases sugar metabolism, impairs tissue integrity and learning ability, reduces honeybee lifespan and foragers homing ability, modulates hormonal stress and innate immunity pathways (mainly the octopamine pathway), and induces hormonal stress, oxidative stress, and energetic stress by using multiple experimental methods, such as real-time quantitative PCR and mass spectrometry and 2-dimensional differential in-gel electrophoresis (Antunez et al., 2009; Mayack and Naug, 2009; Dussaubat et al., 2012; Wolf et al., 2014; Mayack et al., 2015; Piiroinen and Goulson, 2016). Furthermore, a previous study present that no energetic stress is caused by *N. ceranae* infection in *N. ceranae*-tolerant honeybees (Kurze et al., 2016). Therefore, breeding *N. ceranae*-tolerant honeybees may be conducive to decreasing the damage caused by *N. ceranae* infection. Interestingly, with increasing levels of infection with *N. ceranae*, honeybees often choose sunflower honey over honeydew honey or black locust honey. This selection may occur because sunflower honey has higher antimicrobial activity due to its higher H_2_O_2_ concentration (Oelschlaegel et al., 2012; Gherman et al., 2014). Moving honeybee colonies to sunflower-rich sites may be a crucial tactic for healing and improving resistance to nosemosis.

Mites, such as *Tropilaelaps* mites and *V. destructor*, are also familiar parasites in honeybees. Mites cause physiological and physical damage to honeybees by feeding on the its hemolymph, resulting in suppression of honeybee immune function, triggering premature death of pupae, impairing cognitive ability and reducing the nutrient levels (Degrandi-Hoffman and Chen, 2015). *Varroa* mites can transmit bacteria through their connection with worker honeybees in the hive, and enable bacteria to enter the hemolymph when they absorb hemolymph from pupae, which may contribute to bacterial infections, and accelerat their damage to honeybee pupae (Kanbar and Engels, 2003; Gatschenberger et al., 2013). Some subspecies of honeybee are resistant to mites, such as *Apis mellifera* from far-eastern Russia and *Apis mellifera syriaca*. By using next-generation sequencing, proteome analysis and near-infrared cameras, it is found that several factors including inheritance, natural selection, social immunity, higher proportions of drone broods, neuronal and olfactory sensitivity and gene expression regulation are believed to be conducive to their tolerance (Rinderer et al., 2001; Haddad et al., 2016; Hu et al., 2016). Besides, recent research shows that certain doses of α-terpineol can repel female mites from entering into brood cells in hives (Dong et al., 2016), which indicates that α-terpineol may be a potential substance for future use in resisting mites in apiculture.

In addition to *N. ceranae* and mites, the small hive beetle is also a parasitic pest of honeybees. The small hive beetle can weaken and collapse a honeybee colony in a matter of 2 weeks. Gas chromatographic-electroantennographic detection and gas chromatography-mass spectrometric analysis indicate that eight volatiles that are released by worker honeybees, namely, decanal, octanal, nonanal, 2-heptanone, 2-nonanone, hexyl acetate, isopentyl acetate, and isopentyl acetate, are very attractive to the small hive beetle, especially the female small hive beetle. Honeybees release these volatiles more readily under various abiotic or biotic stimuli than in their absence (Wenning, 2001; Torto et al., 2005; Rolff et al., 2009). Thus, decreasing certain type of biotic and abiotic stresses in honeybees may reduce the damage caused by the small hive beetle.

## Abiotic stress in honeybees

In addition to biotic stresses, many abiotic stresses also contribute to the colony collapse in honeybee. Abiotic stress can be triggered by multiple factors, such as temperature, pesticides and nutrition. These stress factors rarely kill honeybees directly. However, the effect of one of these stresses may make honeybees more susceptible to other environmental stresses. Honeybees exhibit different defense responses to various abiotic stresses.

### Temperature

**Figure 3:**
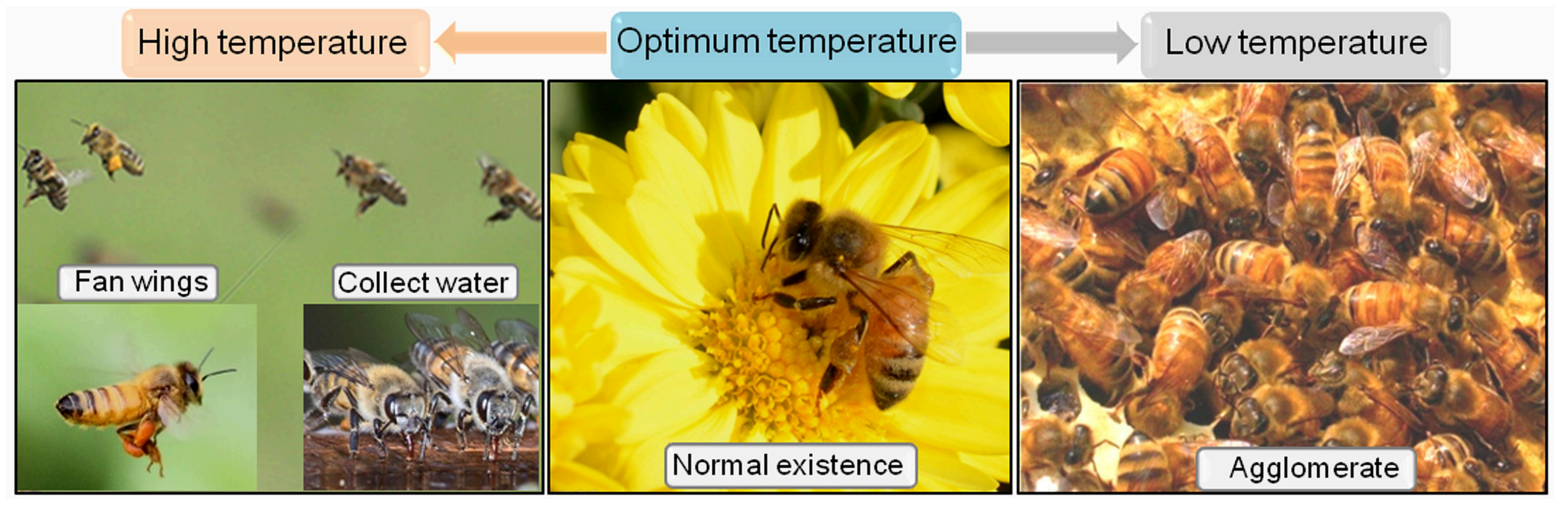
The behavior of honeybee to defense temperature. The honeybees can normal existence under optimum temperature. When exposed to heat, they may fan wings, and collect water to produce convective cooling. When suffered to cold, they may quickly contract, and agglomerate to generate heat cold.

Unsuitable temperature conditions may result in stress in honeybees. The relative humidity of brood comb is maintained at ~70%, and the temperature of brood comb is in the range of 33–36°C. Humidity in the range of 30–75% has no obvious influence on honeybee survival, whereas temperature change has a stronger effect on honeybee survival, and over a long period of time, honeybee will lose the ability to tolerate high or low temperatures (Heinrich, 1979; Southwick and Heldmaier, 1987; Petz et al., 2004). For example, a previous study reports that a noticeable reduction in honeybee survival is observed at higher temperatures (Abou-Shaara et al., 2012). Extreme temperature can trigger temperature stress for foragers, and foragers do not collect nectar and pollen at temperatures that are too hot or too cold (Park et al., 2015). Honeybees regulate their head temperature at a high ambient temperature, and the thoracic temperature is secondarily stabilized. Nevertheless, the thoracic temperature is regulated at low ambient temperatures (Heinrich, 1979). Many other thermoregulatory behaviors can also be performed by honeybees to defend against temperature stress (Figure 3).

When suffering from high temperatures in the honeycomb, honeybees will take many measures to cope with it (Figure 3). First, some honeybees move across the combs, fan their wings to generate air currents, and produce convective cooling to the honeycomb. Second, honeybees will collect more water, and use their proboscises to spread water in a film through tongue lashing, which enables evaporative cooling in combination with the fanning wings of other honeybees. Third, many honeybees will evacuate the hive, possibly to make more room for the honeybees that are responsible for evaporative cooling. Last but not least, reallocation of labor will occur when a honeybee colony is suffering from high temperatures. The additional labor needed to address heat stress may be obtained by the switching of honeybees from any other task and by the activation of the reserve labor of honeybees in the middle-aged caste (Southwick and Heldmaier, 1987; Johnson, 2002; Lindauer and Watkin, 2015). In addition to behavioral changes, Hsps, such as Hsp24.2, Hsp23.0, Hsp27.6, Hsp70, grp78, Hsp 80, and Hsp90 are reported to take part in the high temperature stress response of honeybees by increasing their mRNA or protein levels (Johnson, 2002; Elekonich, 2009; Liu et al., 2012, 2014; Koo et al., 2015). Furthermore, under treatment at 43°C, the transcription of *AccHsp22.6* continues to increase from 1 to 5 h in *Apis cerana cerana*. Knockdown of *AccHsp22.6* significantly reduces survival of *Apis cerana cerana* under heat stress (Zhang et al., 2014). Overexpression of *AccHsp22.6* may increase the tolerance of *Apis cerana cerana* to heat, thus enhance their survival under high temperature.

In situations of cold stress, honeybees usually perform rapid contraction to vibrate their thoracic muscles with the wing immobile, and congregate to produce body heat (Figure 3; Heinrich, 1980). Interestingly, a type of honeybee called hot bees has been found by using a modified brood nest and an infrared-sensitive thermal imaging camera in the honeybee hive. Hot bees do not carry out any other work. Their abdomens exhibit rapid and continuous respiratory movements, and their thorax temperatures can range from 34.1 to 42.5°C. This high thoracic temperature is derived from previous warm-up and heating activity on the surface of the comb. Hot bees can firmly press their thoraxes onto the capped surface of sealed brood cells or remain inside empty cells. Within 30 min, they can enhance the temperature of the brood cap by 3°C through heating the brood cap surface, and increase the temperature of adjacent brood cells by 2.5°C by sitting inside empty cells, leaving a “hot spot” in the place that has been warmed (Bujok et al., 2002; Kleinhenz et al., 2003; Dantas, 2016). Glutaredoxin 1, glutaredoxin 2, thioredoxin 1, TGF-β-activated kinase-1, mitochondrial thioredoxin peroxidase gene 3, and thioredoxin gene 2 have been demonstrated to be associated with cold stress regulation in *Apis cerana cerana* (Meng et al., 2011; Gong et al., 2012; Yao et al., 2013, 2014).

### Pesticides

**Figure 4:**
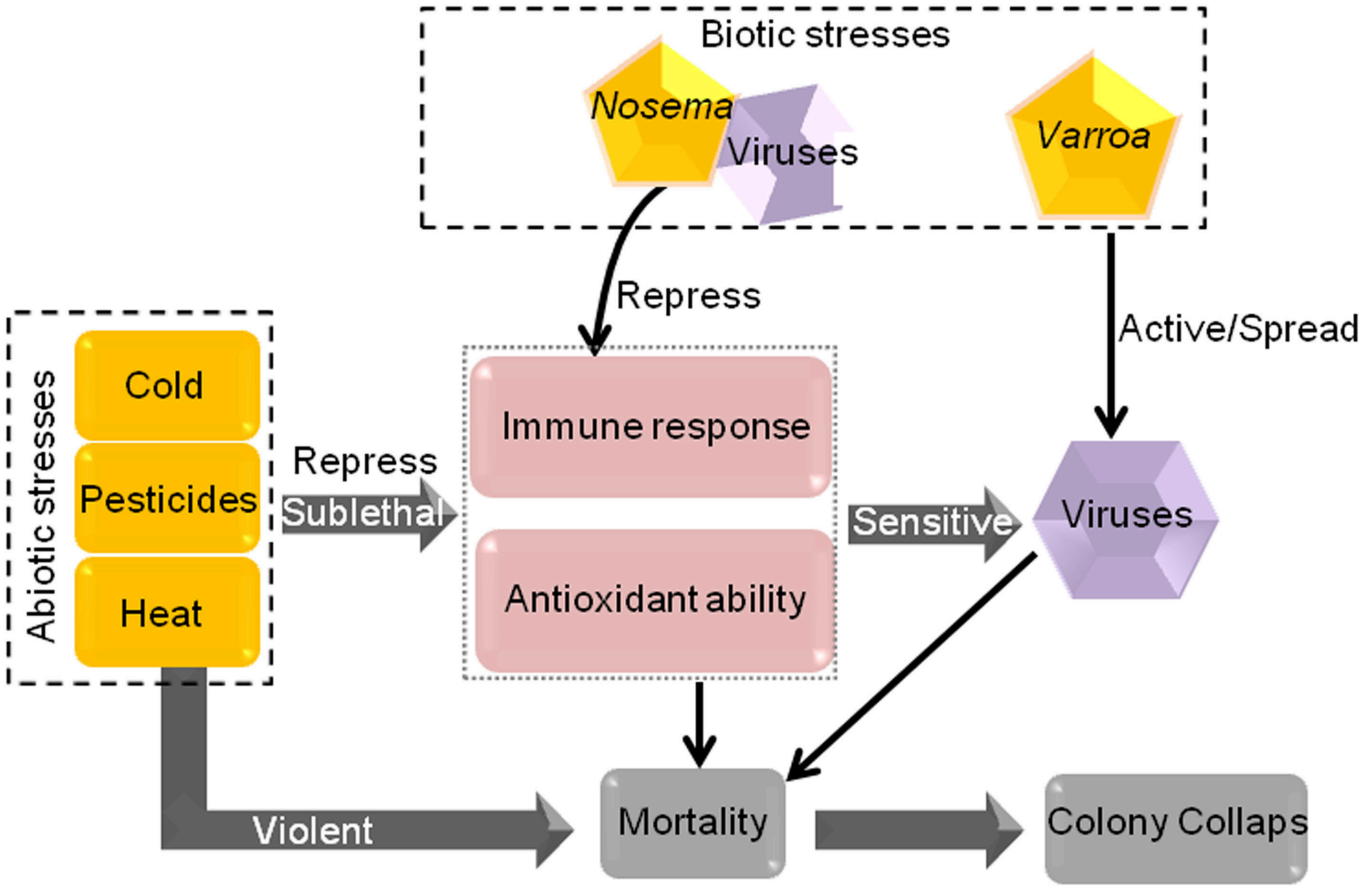
The mode of action of some biotic and abiotic stresses in honeybees. Sublethal cold, pesticides and heat reduce the immune response and antioxidant ability of honeybees, resulting in greater honeybee sensitivity to viral infection. Furthermore, *Varroa* can spread and activate viruses. All of these stress factors will lead to a certain degree of honeybee mortality. Violent abiotic stresses can cause mortality directly. If the mortality progressively increases, colony collapse will occur.

Many pesticides are used in agriculture to reduce crop damage caused by pests and weeds. However, pesticides often trigger considerable damage to honeybees, including influence honeybee behavior, antioxidant ability and immunocompetence, and are linked to honeybee disease through interaction with pathogenic stressors by increasing the sensitivity of honeybees to viral infection (Figure 4; Qiao et al., 2005; Chakrabarti et al., 2015; Kakumanu et al., 2016; Sanchez-Bayo et al., 2016). Though honeybees have been found to lack certain detoxification enzymes used by other insects in response to pesticides (Claudianos et al., 2006), many genes and mechanisms are employed by honeybees to defend against pesticide stresses. Notably, different pesticides may cause diverse degrees of damage to honeybee, and honeybees' defense measures in response to them are not identical.

Recent studies, using radiofrequency identification (RFID) and realistic experiment, show that exposure to a sublethal dose of neonicotinoid pesticide impairs olfactory, learning acquisition, and social immune system, influences the mortality of honeybee workers and egg laying by queens, reduces life span, foraging activity and hygienic behavior, increases forging flights, and causes precocious foraging in honeybees (Schneider et al., 2012; Tsvetkov et al., 2017). Neonicotinoids poison honeybees by the upregulation of a leucine-rich repeat protein (Amel\LRR), then negatively regulating an NF-κB immune signaling pathway and actively promoting the replication of DWV in *Apis mellifera* (Di et al., 2013), and by interacting with odorant-binding protein and chemosensory protein 1 to influence honeybee olfactory function (Li et al., 2015, 2017). Tau-fluvalinate can be metabolized by *CYP9Q1, CYP9Q2*, and *CYP9Q3*, and the metabolite can then undergo further cleavage by carboxylesterases (Mao et al., 2011). Furthermore, analysis of genome-wide expression patterns in honeybee have identified a total of 1,118 differentially expressed transcripts related to immunity, nutrition, detoxification and behavioral maturation as responding to fluvalinate and coumaphos treatment (Schmehl et al., 2014). Many other genes, for example *GSTS1, GSTT1, GSTO2*, Hsp70, grp78, and Hsp90 are also reported that participate in different pesticide stress reaponse (Yan et al., 2013; Zhang et al., 2013; Koo et al., 2015; Liu et al., 2016). In addition to genes that regulate pesticide stress, abscisic acid (ABA) (Glossary) has been found to enhance the tolerance of honeybees to pesticides. Honeybees can tolerate high concentrations of ABA (1 mM) (Negri et al., 2015). ABA supplementation in syrup solution results in improving wound healing and hemocyte response in non-selfrecognition, increasing plasmatocyte, granulocyte activation, and winter honeybee populations. ABA may therefore be used in apiculture to decrease colony losses to some extent.

In addition to the pesticides used in agriculture, some medicines used to kill pathogens in honeybee colonies can also trigger a stress response in honeybees. For example, formic acid that used by beekeepers to kill *Varroa* mites has a negative effect on brood survival, and reduces honeybee longevity by altering the expression of detoxification and development related genes, immune system components and the c-Jun amino-terminal kinase (JNK) pathway (Fries, 1991; Underwood and Currie, 2003; Di et al., 2013). A recent study also shows that eight immunity-related genes contribute to the regulation of acaricide tratment in honeybees (Boncristiani et al., 2012), indicating that these immune response genes may play essential roles in decreasing the damage from acaricide.

### Nutrition

Apart from pesticide and temperature stress, nutrition stress is also an abiotic stress for honeybees. It influences the immunocompetence and the survival of honeybees upon exposure to other stressors, and nutritional stress triggered by the habitat may be among the reasons for the recent colony collapse of honeybees (Naug, 2009; Archer et al., 2013). Honeybees fed polyfloral diets and protein-rich diets possess higher fat body contents, hemocyte concentrations, GOX oxidase activity and phenoloxidase activity, and dietary protein deficiency increases the susceptibility of organisms to multiple diseases (Alaux et al., 2010). Moreover, providing honeybees with high-quality nutrition (pollen-based diet) reduces the sensitivity of honeybees to one-third of pesticides (Schmehl et al., 2014). Therefore, balanced nutrition is vitally important to maintaining healthy and well-fed colonies, especially in a difficult environment (Brodschneider and Crailsheim, 2010). Particularly worth mentioning is that though adult honeybees have fewer ovarioles and smaller bodies when they are starved at the fifth instar larval stage, the survival of adult bees is increased under starvation condition, because they can better maintain blood sugar levels, decrease energy use and suppress metabolic rate (Wang et al., 2014; Wang Y. et al., 2016). The specific molecular mechanism that involved in honeybees addressing nutrition stress is still not fully explored, and further study should focus on this topic.

### Other abiotic stresses

Dietary salt is important for honeybees. Ingestion too much or too little salt may cause stress to honeybees in some degree. Honeybees are often found collecting brackish water, dirty water and seawater, possibly because these water sources contain low salt concentration. Foragers are reluctant to collect onion nectar, which contains ~1.3% potassium. The 0.4–0.75% Na_2_HPO4, 0–1.5% KCl, and 1.5% NaCl are optimal salt concentration for *Apis mellifera* according to the tests from proboscis extension reflex response (Waller et al., 1972; Lau and Nieh, 2016). Artificially supplying honeybees with salt water at optimal concentrations may contribute to the health of honeybee. Nevertheless, very little is known about the specific genes and signaling pathways that participate in salt stress regulation. RNA sequencing and proteome analysis can be used to reveal the specific molecular mechanisms of the impact of salt stress in honeybees in the near future.

Pesticides, cold, heat, and other abiotic stresses, such as heavy metal and ultraviolet radiation stress, all of them can result in the generation of reactive oxygen species in an organism. Low concentration of reactive oxygen species are necessary for organism. However, high concentration of reactive oxygen species will lead to oxidative damage (Finkel and Holbrook, 2000; Boileau et al., 2003; Mates et al., 2010; Ray et al., 2012). Our previous studies demonstrate that at least 80 genes may participate in the oxidative stress response in *Apis cerana cerana*. However, that is not sufficient to reveal the detailed regulatory mechanisms by which honeybees address oxidative damage caused by different environmental stress factors. Chromatin immunoprecipitation (ChIP) sequencing, ChIP-chip, RNA sequencing and proteome sequencing can be used in the future for the rapid identification of many specific genes, transcription factors, proteins regulated by the transcription factor, and signaling pathways that participate in defense oxidative damage in honeybees.

## Conclusion and future directions

Honeybees are likely to encounter various stress-related factors during their lives (Figure 1A). When these stress factors are present in an individual or in the whole colony, honeybees can address the problem through self-mediation by using social behavior and immune response, changing in energy metabolism, physical barriers and many genes and signaling pathway (Figure 1B). If honeybees cannot overcome the stressors, they may succumb to it and even die (Figure 1C; Potts et al., 2010; Goulson et al., 2015; Perry et al., 2015). Moreover, multiple stresses, either alone or in combination, can compromise the health of honeybees and increase colony mortality. Some abiotic stress factors repress the immune response and antioxidative ability of honeybees, render the honeybees more susceptible to parasites and viruses (Figure 4; Anja and Luc, 2008; Gill et al., 2012).

Although the responses of honeybee to environmental stress have been studied for a long time, many outstanding questions remain. The following research directions may be helpful in the future. First, further studies are needed to explore the process of rapid colony collapse. A detailed understanding of the colony failure process will be helpful for finding the most effective strategy to enhance the resilience of colonies. Second, more efforts should be devoted to revealing the mechanisms and rules of the interaction between different stresses. The interaction between different abiotic stresses, between different biotic stresses, and between abiotic and biotic stresses may increase the severity of their effects on the health and survival of honeybees. Third, researchers should continue to identify more possible key genes and signaling pathways that participate in the stress responses of honeybees. In this process, cytobiological, genetic, and molecular biological methods can be used. Fourth, breeding of disease-resistant honeybees is vital for apiculture. Disease-tolerant honeybees found in colonies through natural selection should be expanded. If possible, overexpression of stress resistance genes in honeybees should be performed to obtain transgenic honeybees, or knockdown of stress sensitivity genes in honeybees can be carried out to obtain mutant honeybees, both of which may exhibit improved stress resistance. However, the current challenge is that transgenesis in honeybees is prohibitively difficult. Finally, provision of sufficient nutrition to honeybees by the beekeeper is likely to play an essential role in maintaining the health of colony bees in apiculture. Good nutrition enhances the immunocompetence of honeybees, and improves the ability of the colony to defend against environmental stress (Naug, 2009; Archer et al., 2013; Schmehl et al., 2014).

## Author contributions

GL, BX, and XG: designed of the work. GL, HZ, BX, and XG: drafted the work. HZ, ZL, and HW: created the figures and tables. All authors approved the final version of the manuscript.

### Conflict of interest statement

The authors declare that the research was conducted in the absence of any commercial or financial relationships that could be construed as a potential conflict of interest. The reviewer JT and handling Editor declared their shared affiliation.

## Glossary

**ß-glucan**: ß-Glucan is a heterogeneous group of glucose homopolymers found in algae, plants, fungi, and some bacteria. It can exert strong immune stimulatory activity in many invertebrate and vertebrate species (Soltanian et al., 2009).

**Innate immune response**: The immune response includes acquired immune response and innate immune response. Like all insects, honeybees lack an acquired immune response to defense against microorganisms. Instead, they depend on the innate immune response, which includes the humoral immune response (synthesis of specific antimicrobial peptides), the cellular immune response (wouding healing, nodulation, phagocytosis, and encapsulation) and the activation of prophenoloxidase in response to microbial infection. Prophenoloxidase can be activated by a serine protease cascade, causing transient synthesis of melanin and quinones (Richard et al., 2008; Chan et al., 2009; Masri and Cremer, 2014). Furthermore, recent study shows that due to these immune characteristics, honeybees may become a potential vivo system for screening antiviral compounds under certain experimental contexts (Fedorova et al., 2011).

**Abscisic acid (ABA)**: ABA is a phytohormone that participates in regulation of fundamental physiological functions in plants. The presence of ABA in honey and nectar has been unambiguously demonstrated (Ferreres et al., 1996; Adie et al., 2007).

## References

[B1] Abou-Shaara H. F. Al-Ghamdi A. A. Mohamed A. A. (2012). Tolerance of two honey bee races to various temperature and relative humidity gradients. Environ. Exp. Biol. 10, 133–138.

[B2] Adie B. A. Perez-Perez J. Perez-Perez M. M. Godoy M. Sanchez-Serrano J. J. Schmelz E. A.. (2007). ABA is an essential signal for plant resistance to pathogens affecting JA biosynthesis and the activation of defenses in Arabidopsis. Plant Cell. 19, 1665–1681. 10.1105/tpc.106.048041 17513501 PMC1913739

[B3] Alaux C. Ducloz F. Crauser D. Le Conte Y. (2010). Diet effects on honeybee immunocompetence. Biol. Lett. 6, 562–565. 10.1098/rsbl.2009.0986 20089536 PMC2936196

[B4] Albert S. Gatschenberger H. Azzami K. Gimple O. Grimmer G. Sumner S.. (2011). Evidence of a novel immune responsive protein in the Hymenoptera. Insect Biochem. Mol. Biol. 41, 968–981. 10.1016/j.ibmb.2011.09.006 22001069

[B5] Ali M. A. M. (2007). Efficacy of certain plant extracts against stonebrood pathogen (*Aspergillus flavus*) in honeybee *in vitro*. Egypt. J. Pest Control 17, 13–19.

[B6] Anja C. Luc D. M. (2008). Synergistic, antagonistic and additive effects of multiple stressors: predation threat, parasitism and pesticide exposure in daphnia magna J. Appl. Ecol. 45, 1820–1828. 10.1111/j.1365-2664.2008.01566.x

[B7] Antunez K. Martin-Hernandez R. Prieto L. Meana A. Zunino P. Higes M. (2009). Immune suppression in the honey bee (*Apis mellifera*) following infection by *Nosema ceranae* (Microsporidia). Environ. Microbiol. 11, 2284–2290. 10.1111/j.1462-2920.2009.01953.x 19737304

[B8] Archer C. R. Pirk C. W. W. Wright G. A. Nicolson S. W. (2013). Nutrition affects survival in African honeybees (*Apis mellifera scutellata*) exposed to interacting stressors. Funct. Ecol. 28, 913–923. 10.1111/1365-2435.12226

[B9] Aronstein K. A. Murray K. D. (2010). Chalkbrood disease in honey bees. J. Invertebr. Pathol. 103(Suppl. 1), S20–S29. 10.1016/j.jip.2009.06.018 19909969

[B10] Azzami K. Ritter W. Tautz J. Beier H. (2012). Infection of honey bees with acute bee paralysis virus does not trigger humoral or cellular immune responses. Arch. Virol. 157, 689–702. 10.1007/s00705-012-1223-0 22258854 PMC3314816

[B11] Bailey L. (1981). Honey Bee Pathology. Sebastopol, CA: Academic press, 13.

[B12] Bailey L. Ball B. V. (1991). Index-Honey Bee Pathology, 2nd Edn. London: Academic press, 185–193.

[B13] Bailey L. Fernando E. F. W. (2010). Effects of sacbrood virus on adult honey-bees. Ann. Appl. Biol. 72, 27–35. 10.1111/j.1744-7348.1972.tb01268.x

[B14] Bailey L. Woods R. D. (1974). Three previously undescribed viruses from the honey bee. J. Gen. Virol. 25, 175–186. 10.1099/0022-1317-25-2-175 4215869

[B15] Boileau T. W. Bray T. M. Bomser J. A. (2003). Ultraviolet radiation modulates nuclear factor kappa B activation in human lens epithelial cells. J. Biochem. Mol. Toxicol. 17, 108–113. 10.1002/jbt.10067 12717744

[B16] Boncristiani H. Underwood R. Schwarz R. Evans J. D. Pettis J. vanEngelsdorp D. (2012). Direct effect of acaricides on pathogen loads and gene expression levels in honey bees *Apis mellifera*. J. Insect Physiol. 58, 613–620. 10.1016/j.jinsphys.2011.12.011 22212860

[B17] Boncristiani H. F. Evans J. D. Chen Y. Pettis J. Murphy C. Lopez D. L. (2013). *In vitro* infection of pupae with Israeli acute paralysis virus suggests disturbance of transcriptional homeostasis in honey bees (*Apis mellifera*). PLoS ONE 8:e73429 10.1371/journal.pone.0073429 24039938 PMC3764161

[B18] Booton R. D. Iwasa Y. J. Marshall A. R. Childs D. Z. (2017). Stress-mediated Allee effects can cause the sudden collapse of honey bee colonies. J. Theor. Biol. 420, 213–219. 10.1016/j.jtbi.2017.03.009 28288794

[B19] Brodschneider R. Crailsheim K. (2010). Nutrition and health in honey bees. Apidologie 41, 278–294. 10.1051/apido/2010012

[B20] Brødsgaard C. J. Ritter W. Hansen H. (1998). Response of *in vitro* reared honey bee larvae to various doses of *Paenibacillus larvae* larvae spores. Apidologie 29, 569–578. 10.1051/apido:19980609

[B21] Bujok B. Kleinhenz M. Fuchs S. Tautz J. (2002). Hot spots in the bee hive. Naturwissenschaften 89, 299–301. 10.1007/s00114-002-0338-7 12216858

[B22] Chaimanee V. Thongtue U. Sornmai N. Songsri S. Pettis J. S. (2017). Antimicrobial activity of plant extracts against the honeybee pathogens, *Paenibacillus larvae* and *Ascosphaera apis* and their topical toxicity to *Apis mellifera* adults. J. Appl. Microbiol.. 123, 1160–1167. 10.1111/jam.13579 28869798

[B23] Chakrabarti P. Rana S. Sarkar S. Smith B. Basu P. (2015). Pesticide-induced oxidative stress in laboratory and field populations of native honey bees along intensive agricultural landscapes in two eastern indian states. Apidologie 46, 107–129. 10.1007/s13592-014-0308-z

[B24] Chan Q. W. Foster L. J. (2008). Changes in protein expression during honey bee larval development. Genome Biol. 9:R156. 10.1186/gb-2008-9-10-r156 18959778 PMC2760883

[B25] Chan Q. W. Melathopoulos A. P. Pernal S. F. Foster L. J. (2009). The innate immune and systemic response in honey bees to a bacterial pathogen, *Paenibacillus larvae*. BMC Genomics. 10:387. 10.1186/1471-2164-10-387 19695106 PMC2907699

[B26] Chen D. Guo R. Xu X. Xiong C. Liang Q. Zheng Y.. (2017). Uncovering the immune responses of *Apis mellifera ligustica* larval gut to *Ascosphaera apis* infection utilizing transcriptome sequencing. Gene 621, 40–50. 10.1016/j.gene.207.04.022 28427951

[B27] Chen X. Y. (2013a). Harm and control of chalkbrood disease and *Aspergillus flavus* disease. J. Bee. 33:24.

[B28] Chen X. Y. (2013b). Several control methods of bee mite. J. Bee. 33:35.

[B29] Chen Y. Pettis J. S. Feldlaufer M. F. (2005). Detection of multiple viruses in queens of the honey bee *Apis mellifera* L. J. Invertebr. Pathol. 90, 118–121. 10.1016/j.jip.2005.08.005 16214161

[B30] Clark T. B. Whitcomb R. F. Tully J. G. Mouches C. Saillard C. Bové J. (1985). *Spiroplasma melliferum*, a new species from the honeybee (*Apis mellifera*). Int. J. Syst. Bacteriol. 35, 296–308. 10.1099/00207713-35-3-296

[B31] Claudianos C. Ranson H. Johnson R. M. Biswas S. Schuler M. A. Berenbaum M. R.. (2006). A deficit of detoxification enzymes: pesticide sensitivity and environmental response in the honeybee. Insect Mol. Biol. 15, 615–636. 10.1111/j.1365-2583.2006.00672.x 17069637 PMC1761136

[B32] Collison E. Hird H. Cresswell J. Tyler C. (2016). Interactive effects of pesticide exposure and pathogen infection on bee health - a critical analysis. Biol. Rev. Camb. Philos. Soc. 91, 1006–1019. 10.1111/brv.12206 26150129

[B33] Cox-Foster D. L. Conlan S. Holmes E. C. Palacios G. Evans J. D. Moran N. A.. (2007). A metagenomic survey of microbes in honey bee colony collapse disorder. Science 318, 283–287. 10.1126/science.1146498 17823314

[B34] Cremer S. Armitage S. A. Schmid-Hempel P. (2007). Social immunity. Curr. Biol. 17, R693–R702. 10.1016/j.cub.2007.06.008 17714663

[B35] Dainat B. Evans J. D. Chen Y. P. Gauthier L. Neumann P. (2012). Dead or alive: deformed wing virus and *Varroa destructor* reduce the life span of winter honeybees. Appl. Environ. Microbiol. 78, 981–987. 10.1128/AEM.06537-11 22179240 PMC3273028

[B36] Dantas M. (2016). Thermogenesis in stingless bees: an approach with emphasis on brood's thermal contribution. J. Anim. Behav. Biometeorol. 4, 101–108. 10.14269/2318-1265/jabb.v4n4p101-108

[B37] Degrandi-Hoffman G. Chen Y. (2015). Nutrition, immunity and viral infections in honey bees. Curr. Opin. Insect Sci. 10, 170–176. 10.1016/j.cois.2015.05.007 29588005

[B38] de Miranda J. R. Cordoni G. Budge G. (2010). The Acute bee paralysis virus-Kashmir bee virus-Israeli acute paralysis virus complex. J. Invertebr. Pathol. 103(Suppl. 1), S30–S47. 10.1016/j.jip.2009.06.014 19909972

[B39] Di P. G. Cavaliere V. Annoscia D. Varricchio P. Caprio E. Nazzi F. (2013). Neonicotinoid clothianidin adversely affects insect immunity and promotes replication of a viral pathogen in honey bees. Proc. Natl. Acad. Sci. U.S.A. 110, 18466–18471. 10.1073/pnas.1314923110 24145453 PMC3831983

[B40] Di Prisco G. Pennacchio F. Caprio E. Boncristiani H. F. Jr. Evans J. D. Chen Y. (2011). *Varroa destructor* is an effective vector of Israeli acute paralysis virus in the honeybee, *Apis mellifera*. J. Gen. Virol. 92, 151–155. 10.1099/vir.0.023853-0 20926637

[B41] Dong X. Kashio M. Peng G. Wang X. Tominaga M. Kadowaki T. (2016). Isoform-specific modulation of the chemical sensitivity of conserved TRPA1 channel in the major honeybee ectoparasitic mite, *Tropilaelaps mercedesae*. Open Biol. 6:160042. 10.1098/rsob.160042 27307515 PMC4929936

[B42] Dussaubat C. Brunet J. L. Higes M. Colbourne J. K. Lopez J. Choi J. H.. (2012). Gut pathology and responses to the microsporidium *Nosema ceranae* in the honey bee *Apis mellifera*. PLoS ONE 7:e37017. 10.1371/journal.pone.0037017 22623972 PMC3356400

[B43] Elekonich M. M. (2009). Extreme thermotolerance and behavioral induction of 70-kDa heat shock proteins and their encoding genes in honey bees. Cell Stress Chaperones 14, 219–226. 10.1007/s12192-008-0063-z 18696260 PMC2727992

[B44] Ellis J. D. Munn P. A. (2005). The worldwide health status of honey bees. Bee World. 86, 88–101. 10.1080/0005772X.2005.11417323

[B45] Engel M. S. (2001). A monography of the Baltic amber bees and evolution of the Apoidea (Hymenoptera). Bull. Am. Mus. Nat. Hist. 35, 1–192. 10.1206/0003-0090(2001)259<0001:AMOTBA>2.0.CO;2

[B46] Evans J. D. Aronstein K. Chen Y. P. Hetru C. Imler J. L. Jiang H.. (2006). Immune pathways and defence mechanisms in honey bees *Apis mellifera*. Insect Mol. Biol. 15, 645–656. 10.1111/j.1365-2583.2006.00682.x 17069638 PMC1847501

[B47] Evans J. D. Schwarz R. S. (2011). Bees brought to their knees: microbes affecting honey bee health. Trends Microbiol. 19, 614–620. 10.1016/j.tim.2011.09.003 22032828

[B48] Fedorova A. A. Azzami K. Ryabchikova E. I. Spitsyna Y. E. Silnikov V. N. Ritter W.. (2011). Inactivation of a non-enveloped RNA virus by artificial ribonucleases: honey bees and acute bee paralysis virus as a new experimental model for *in vivo* antiviral activity assessment. Antiviral Res. 91, 267–277. 10.1016/j.antiviral.2011.06.011 21722669

[B49] Ferreres F. Andrade P. Tomás-Barberán F. (1996). Natural occurrence of abscisic acid in heather honey and floral nectar. J. Agric. Food Chem. 44, 2053–2056. 10.1021/jf9507553

[B50] Finkel T. Holbrook N. J. (2000). Oxidants, oxidative stress and the biology of ageing. Nature. 408, 239–247. 10.1038/35041687 11089981

[B51] Flores J. M. Spivak M. Gutiérrez I. (2005). Spores of ascosphaera apis contained in wax foundation can infect honeybee brood. Vet. Microbiol.. 108, 141–144. 10.1016/j.vetmic.2005.03.005 15917141

[B52] Fries I. (1991). Treatment of sealed honey bee brood with formic acid for control of *Varroa jacobsoni*. Am. Bee J. 131, 313–314.

[B53] Gatschenberger H. Azzami K. Tautz J. Beier H. (2013). Antibacterial immune competence of honey bees (*Apis mellifera*) is adapted to different life stages and environmental risks. PLoS ONE. 8:e66415. 10.1371/journal.pone.0066415 23799099 PMC3684586

[B54] Gatschenberger H. Gimple O. Tautz J. Beier H. (2012). Honey bee drones maintain humoral immune competence throughout all life stages in the absence of vitellogenin production. J. Exp. Biol. 215, 1313–1322. 10.1242/jeb.065276 22442369

[B55] Genersch E. Ohe W. V. D. Kaatz H. Schroeder A. Otten C. Büchler R. (2010). The german bee monitoring project: a long term study to understand periodically high winter losses of honey bee colonies. Apidologie 41, 332–352. 10.1051/apido/2010014

[B56] Gherman B. I. Denner A. Bobi,ş O. Dezmirean D. S. Mărghita,ş L. A. Schlüns H. (2014). Pathogen-associated self-medication behavior in the honeybee *Apis mellifera*. Behav. Ecol. Sociobiol. 68, 1777–1784. 10.1007/s00265-014-1786-8

[B57] Gill R. J. Ramos-Rodriguez O. Raine N. E. (2012). Combined pesticide exposure severely affects individual- and colony-level traits in bees. Nature 491, 105–108. 10.1038/nature11585 23086150 PMC3495159

[B58] Gong H. R. Chen X. X. Chen Y. P. Hu F. L. Zhang J. L. Lin Z. G.. (2016). Evidence of *Apis cerana* sacbrood virus infection in *Apis mellifera*. Appl. Environ. Microbiol. 82, 2256–2262. 10.1128/AEM.03292-15 26801569 PMC4959495

[B59] Gong Z. Guo X. Xu B. (2012). Molecular cloning, characterisation and expression of methionine sulfoxide reductase a gene from *Apis cerana cerana*. Apidologie 43, 182–194. 10.1007/s13592-011-0099-4

[B60] Goulson D. Nicholls E. Botias C. Rotheray E. L. (2015). Bee declines driven by combined stress from parasites, pesticides, and lack of flowers. Science 347:1255957. 10.1126/science.1255957 25721506

[B61] Gregorc A. Bowen I. D. (1999). *In situ* localization of heat-shock and histone proteins in honey-bee (*Apis mellifera* l.) larvae infected with *paenibacillus larvae*. Cell Biol. Int. 23, 211–218. 10.1006/cbir.1999.0344 10562442

[B62] Haddad N. Mahmud Batainh A. Suleiman Migdadi O. Saini D. Krishnamurthy V. Parameswaran S.. (2016). Next generation sequencing of *Apis mellifera* syriaca identifies genes for Varroa resistance and beneficial bee keeping traits. Insect Sci. 23, 579–590. 10.1111/1744-7917.12205 25615619

[B63] Han B. Zhang L. Feng M. Fang Y. Li J. (2013). An integrated proteomics reveals pathological mechanism of honeybee (*Apis cerena*) sacbrood disease. J. Proteome Res. 12, 1881–1897. 10.1021/pr301226d 23418745

[B64] Heinrich B. (1979). Keeping a cool head: honeybee thermoregulation. Science 205, 1269–1271. 10.1126/science.205.4412.1269 17750151

[B65] Heinrich B. (1980). Mechanisms of body-temperature regulation in honeybees, *Apis mellifera*. II. Regulation of thoracic temperature at high air temperatures. J. Exp. Biol. 85, 73–87.

[B66] Highfield A. C. El Nagar A. Mackinder L. C. Noel L. M. Hall M. J. Martin S. J.. (2009). Deformed wing virus implicated in overwintering honeybee colony losses. Appl. Environ. Microbiol. 75, 7212–7220. 10.1128/AEM.02227-09 19783750 PMC2786540

[B67] Hou C. Rivkin H. Slabezki Y. Chejanovsky N. (2014). Dynamics of the presence of israeli acute paralysis virus in honey bee colonies with colony collapse disorder. Viruses 6, 2012–2027. 10.3390/v6052012 24800677 PMC4036543

[B68] Hu H. Bienefeld K. Wegener J. Zautke F. Hao Y. Feng M.. (2016). Proteome analysis of the hemolymph, mushroom body, and antenna provides novel insight into honeybee resistance against Varroa infestation. J. Proteome Res. 15, 2841–2854. 10.1021/acs.jproteome.6b00423 27384112

[B69] Johnson B. R. (2002). Reallocation of labor in honeybee colonies during heat stress: the relative roles of task switching and the activation of reserve labor. Behav. Ecol. Sociobiol. 51, 188–196. 10.1007/s00265-001-0419-1

[B70] Kakumanu M. L. Reeves A. M. Anderson T. D. Rodrigues R. R. Williams M. A. (2016). Honey bee gut microbiome is altered by in-hive pesticide exposures. Front. Microbiol. 7:1255. 10.3389/fmicb.2016.01255 27579024 PMC4985556

[B71] Kanbar G. Engels W. (2003). Ultrastructure and bacterial infection of wounds in honey bee (*Apis mellifera*) pupae punctured by Varroa mites. Parasitol. Res. 90:438 10.1007/s00436-003-0899-1 27520624

[B72] Kerr J. T. Pindar A. Galpern P. Packer L. Potts S. G. Roberts S. M.. (2015). Climate change. Climate change impacts on bumblebees converge across continents. Science 349, 177–180. 10.1126/science.aaa7031 26160945

[B73] Klein A. M. Vaissiere B. E. Cane J. H. Steffan-Dewenter I. Cunningham S. A. Kremen C.. (2007). Importance of pollinators in changing landscapes for world crops. Proc. Biol. Sci. 274, 303–313. 10.1098/rspb.2006.3721 17164193 PMC1702377

[B74] Kleinhenz M. Bujok B. Fuchs S. Tautz J. (2003). Hot bees in empty broodnest cells: heating from within. J. Exp. Biol. 206, 4217–4231. 10.1242/jeb.00680 14581592

[B75] Koo J. Son T. G. Kim S. Y. Lee K. Y. (2015). Differential responses of *Apis mellifera*, heat shock protein genes to heat shock, flower-thinning formulations, and imidacloprid. J. Asia. Pac. Entomol. 18, 583–589. 10.1016/j.aspen.2015.06.011

[B76] Kulhanek K. Steinhauer N. Rennich K. Caron D. M. Sagili R. R. Pettis J. S. (2017). A national survey of managed honey bee 2015–2016 annual colony losses in the USA. J. Apic. Res. 56, 328–340. 10.1080/00218839.2017.1344496

[B77] Kurze C. Mayack C. Hirche F. Stangl G. I. Le Conte Y. Kryger P.. (2016). *Nosema* spp. infections cause no energetic stress in tolerant honeybees. Parasitol. Res. 115, 2381–2388. 10.1007/s00436-016-4988-3 26976406

[B78] Lau P. W. Nieh J. C. (2016). Salt preferences of honey bee water foragers. J. Exp. Biol. 219, 790–796. 10.1242/jeb.132019 26823100

[B79] Lemaitre B. Hoffmann J. (2007). The host defense of *Drosophila melanogaster*. Annu. Rev. Immunol. 25, 697–743. 10.1146/annurev.immunol.25.022106.141615 17201680

[B80] Li G. Jia H. Wang H. Yan Y. Guo X. Sun Q.. (2016a). A typical RNA-binding protein gene (AccRBM11) in *Apis cerana cerana*: characterization of AccRBM11 and its possible involvement in development and stress responses. Cell Stress Chaperones 21, 1005–1019. 10.1007/s12192-016-0725-1 27590229 PMC5083670

[B81] Li G. Zhao H. Wang H. Guo X. Guo X. Sun Q.. (2016b). Characterization of a *Decapentapletic Gene* (AccDpp) from *Apis cerana cerana* and its possible involvement in development and response to oxidative stress. PLoS ONE 11:e0149117. 10.1371/journal.pone.0149117 26881804 PMC4755538

[B82] Li H. Tan J. Song X. Wu F. Tang M. Hua Q.. (2017). Sublethal doses of neonicotinoid imidacloprid can interact with honey bee chemosensory protein 1 (CSP1) and inhibit its function. Biochem. Biophys. Res. Commun. 486, 391–397. 10.1016/j.bbrc.2017.03.051 28315331

[B83] Li H. Wu F. Zhao L. Tan J. Jiang H. Hu F. (2015). Neonicotinoid insecticide interact with honeybee odorant-binding protein: implication for olfactory dysfunction. Int. J. Biol. Macromol. 81, 624–630. 10.1016/j.ijbiomac.2015.08.055 26318218

[B84] Li J. L. Cornman R. S. Evans J. D. Pettis J. S. Zhao Y. Murphy C.. (2014). Systemic spread and propagation of a plant-pathogenic virus in European honeybees, *Apis mellifera*. MBio 5, e00898–e00813. 10.1128/mBio.00898-13 24449751 PMC3903276

[B85] Lindauer M. Watkin M. B. (2015). Division of labor in the honeybee colony. Bee World. 34, 85–90. 10.1080/0005772X.1953.11094792

[B86] Liu S. Liu F. Jia H. Yan Y. Wang H. Guo X.. (2016). A glutathione S-transferase gene associated with antioxidant properties isolated from *Apis cerana cerana*. Naturwissenschaften. 103:43. 10.1007/s00114-016-1362-3 27126403

[B87] Liu X. Zhang Y. Yan X. Han R. (2010). Prevention of Chinese sacbrood virus infection in *Apis cerana* using RNA interference. Curr. Microbiol. 61, 422–428. 10.1007/s00284-010-9633-2 20379718

[B88] Liu Z. Xi D. Kang M. Guo X. Xu B. (2012). Molecular cloning and characterization of Hsp27.6: the first reported small heat shock protein from *Apis cerana cerana*. Cell Stress Chaperones 17, 539–551. 10.1007/s12192-012-0330-x 22351490 PMC3535166

[B89] Liu Z. Yao P. Guo X. Xu B. (2014). Two small heat shock protein genes in *Apis cerana cerana*: characterization, regulation, and developmental expression. Gene 545, 205–214. 10.1016/j.gene.2014.05.034 24835315

[B90] Mao W. Schuler M. A. Berenbaum M. R. (2011). CYP9Q-mediated detoxification of acaricides in the honey bee (*Apis mellifera*). Proc. Natl. Acad. Sci. U.S.A. 108, 12657–12662. 10.1073/pnas.1109535108 21775671 PMC3150950

[B91] Maori E. Lavi S. Mozes-Koch R. Gantman Y. Peretz Y. Edelbaum O.. (2007). Isolation and characterization of Israeli acute paralysis virus, a dicistrovirus affecting honeybees in Israel: evidence for diversity due to intra- and inter-species recombination. J. Gen. Virol. 88, 3428–3438. 10.1099/vir.0.83284-0 18024913

[B92] Maori E. Paldi N. Shafir S. Kalev H. Tsur E. Glick E.. (2009). IAPV, a bee-affecting virus associated with colony collapse disorder can be silenced by dsRNA ingestion. Insect Mol. Biol. 18, 55–60. 10.1111/j.1365-2583.2009.00847.x 19196347

[B93] Martin S. J. (2001). *Varroa destructor* reproduction during the winter in *Apis mellifera* colonies in UK. Exp. Appl. Acarol. 25, 321–325. 10.1023/A:1017943824777 11603739

[B94] Martin S. J. Highfield A. C. Brettell L. Villalobos E. M. Budge G. E. Powell M.. (2012). Global honey bee viral landscape altered by a parasitic mite. Science 336, 1304–1306. 10.1126/science.1220941 22679096

[B95] Masri L. Cremer S. (2014). Individual and social immunisation in insects. Trends Immunol. 35, 471–482. 10.1016/j.it.2014.08.005 25245882

[B96] Mates J. M. Segura J. A. Alonso F. J. Marquez J. (2010). Roles of dioxins and heavy metals in cancer and neurological diseases using ROS-mediated mechanisms. Free Radic. Biol. Med. 49, 1328–1341. 10.1016/j.freeradbiomed.2010.07.028 20696237

[B97] Mayack C. Natsopoulou M. E. McMahon D. P. (2015). Nosema ceranae alters a highly conserved hormonal stress pathway in honeybees. Insect Mol. Biol. 24, 662–670. 10.1111/imb.12190 26335565

[B98] Mayack C. Naug D. (2009). Energetic stress in the honeybee *Apis mellifera* from *Nosema ceranae* infection. J. Invertebr. Pathol. 100, 185–188. 10.1016/j.jip.2008.12.001 19135448

[B99] Mazzei M. Fronte B. Sagona S. Carrozza M. L. Forzan M. Pizzurro F.. (2016). Effect of 1,3-1,6 β-glucan on natural and experimental deformed wing virus infection in newly emerged honeybees (*Apis mellifera ligustica*). PLoS ONE 11:e0166297. 10.1371/journal.pone.0166297 27829027 PMC5102454

[B100] Mehr A. A. Sackett W. T. Wilson R. R. (1978). Persistence of chalkbrood (*Ascosphaera apis*) in some North American honeybee colonies one year after infection. Apiacta 13, 99–102.

[B101] Meng F. Kang M. Liu L. Luo L. Xu B. Guo X. (2011). Characterization of the TAK1 gene in *Apis cerana cerana* (AccTAK1) and its involvement in the regulation of tissue-specific development. BMB Rep. 44, 187–192. 10.5483/BMBRep.2011.44.3.187 21429297

[B102] Meng F. Zhang Y. Liu F. Guo X. Xu B. (2014). Characterization and mutational analysis of omega-class GST (GSTO1) from *Apis cerana* cerana, a gene involved in response to oxidative stress. PLoS ONE 9:e93100. 10.1371/journal.pone.0093100 24667966 PMC3965517

[B103] Michaud S. Boncristiani H. F. Jr. Gouw J. W. Strand M. K. Pettis J. Rueppell O. (2014). Response of the honey bee (*Apis mellifera*) proteome to israeli acute p. Can. J. Zool. 93, 711–720. 10.1139/cjz-2014-0181

[B104] Moeller F. E. (1956). The behaviour of nosema-infected bees affecting their position in the winter cluster. J. Econ. Entomol. 49, 743–745. 10.1093/jee/49.6.743

[B105] Morse R. A. Nowogrodzki R. (1978). Honey Bee Pests, Predators, and Diseases. Ithaca; London: Associates a division of Cornell University Press; Comstock Pub.

[B106] Mouches C. Bove J. M. Tully J. G. Rose D. L. McCoy R. E. Carle-Junca P.. (1983). *Spiroplasma apis*, a new species from the honey-bee *Apis mellifera*. Ann. Microbiol. 134A, 383–397. 6195951

[B107] Naug D. (2009). Nutritional stress due to habitat loss may explain recent honeybee colony collapses. Biol. Conserv. 142, 2369–2372. 10.1016/j.biocon.2009.04.007

[B108] Negri P. Maggi M. D. Ramirez L. Feudis L. D. Szwarski N. Quintana S. (2015). Abscisic acid enhances the immune response in *Apis mellifera*, and contributes to the colony fitness. Apidologie 46, 1–16. 10.1007/s13592-014-0345-7

[B109] Oelschlaegel S. Pieper L. Staufenbiel R. Gruner M. Zeippert L. Pieper B.. (2012). Floral markers of cornflower (*Centaurea cyanus*) honey and its peroxide antibacterial activity for an alternative treatment of digital dermatitis. J. Agric. Food Chem. 60, 11811–11820. 10.1021/jf303699t 23140532

[B110] Park C. Kang H. S. Jeong J. Kang I. Choi K. Yoo M. S.. (2016). *In-situ* hybridization for the detection of sacbrood virus in infected larvae of the honey bee (*Apis cerana*). J. Comp. Pathol. 154, 258–262. 10.1016/j.jcpa.2015.12.003 26852344

[B111] Park D. Jung J. W. Choi B. S. Jayakodi M. Lee J. Lim J.. (2015). Uncovering the novel characteristics of Asian honey bee, Apis cerana, by whole genome sequencing. BMC Genomics. 16:1. 10.1186/1471-2164-16-1 25553907 PMC4326529

[B112] Perry C. J. Søvik E. Myerscough M. R. Barron A. B. (2015). From the cover: rapid behavioral maturation accelerates failure of stressed honey bee colonies. Proc. Natl. Acad. Sci. U.S.A. 112, 3427 10.1073/pnas.1422089112 25675508 PMC4371971

[B113] Petz M. Stabentheiner A. Crailsheim K. (2004). Respiration of individual honeybee larvae in relation to age and ambient temperature. J. Comp. Physiol. B Biochem. Syst. Environ. Physiol. 174, 511–518. 10.1007/s00360-004-0439-z 15278398

[B114] Piiroinen S. Goulson D. (2016). Chronic neonicotinoid pesticide exposure and parasite stress differentially affects learning in honeybees and bumblebees. Proc. Biol. Sci. 283:20160246. 10.1098/rspb.2016.0246 27053744 PMC4843659

[B115] Potts S. G. Biesmeijer J. C. Kremen C. Neumann P. Schweiger O. Kunin W. E. (2010). Global pollinator declines: trends, impacts and drivers. Trends Ecol. Evol. 25, 345–353. 10.1016/j.tree.2010.01.007 20188434

[B116] Qiao D. Seidler F. J. Slotkin T. A. (2005). Oxidative mechanisms contributing to the developmental neurotoxicity of nicotine and chlorpyrifos. Toxicol. Appl. Pharmacol. 206, 17–26. 10.1016/j.taap.2004.11.003 15963341

[B117] Randolt K. Gimple O. Geissendorfer J. Reinders J. Prusko C. Mueller M. J.. (2008). Immune-related proteins induced in the hemolymph after aseptic and septic injury differ in honey bee worker larvae and adults. Arch. Insect Biochem. Physiol. 69, 155–167. 10.1002/arch.20269 18979500

[B118] Ratnieks F. L. Carreck N. L. (2010). Ecology. Clarity on honey bee collapse? Science 327, 152–153. 10.1126/science.1185563 20056879

[B119] Ray P. D. Huang B. W. Tsuji Y. (2012). Reactive oxygen species (ROS) homeostasis and redox regulation in cellular signaling. Cell. Signal. 24, 981–990. 10.1016/j.cellsig.2012.01.008 22286106 PMC3454471

[B120] Richard F. J. Aubert A. Grozinger C. M. (2008). Modulation of social interactions by immune stimulation in honey bee, *Apis mellifera*, workers. BMC Biol. 6:50. 10.1186/1741-7007-6-50 19014614 PMC2596086

[B121] Richard F. J. Holt H. L. Grozinger C. M. (2012). Effects of immunostimulation on social behavior, chemical communication and genome-wide gene expression in honey bee workers (*Apis mellifera*). BMC Genomics 13:558. 10.1186/1471-2164-13-558 23072398 PMC3483235

[B122] Rinderer T. E. De Guzman L. I. Delatte G. T. Stelzer J. A. Lancaster V. A. Kuznetsov V. (2001). Resistance to the parasitic mite varroa destructor in honey bees from far-eastern russia. Apidologie 32, 381–394. 10.1051/apido:2001138

[B123] Rolff J. Reynolds S. Imler J. L. Eleftheriano I. (2009). Insect Infection and Immunity: Evolution, Ecology and Mechanisms. New York, NY: Oxford University Press.

[B124] Rueppell O. Hayworth M. K. Ross N. P. (2010). Altruistic self-removal of health-compromised honey bee workers from their hive. J. Evol. Biol. 23, 1538–1546. 10.1111/j.1420-9101.2010.02022.x 20500363

[B125] Runckel C. Flenniken M. L. Engel J. C. Ruby J. G. Ganem D. Andino R.. (2011). Temporal analysis of the honey bee microbiome reveals four novel viruses and seasonal prevalence of known viruses, Nosema, and Crithidia. PLoS ONE 6:e20656. 10.1371/journal.pone.0020656 21687739 PMC3110205

[B126] Sak B. Sakova K. Ditrich O. (2004). Effects of a novel anti-exospore monoclonal antibody on microsporidial development *in vitro*. Parasitol. Res. 92, 74–80. 10.1007/s00436-003-0988-1 14610668

[B127] Sanchez-Bayo F. Goulson D. Pennacchio F. Nazzi F. Goka K. Desneux N. (2016). Are bee diseases linked to pesticides? - A brief review. Environ. Int. 89–90, 7–11. 10.1016/j.envint.2016.01.009 26826357

[B128] Schmehl D. R. Teal P. E. Frazier J. L. Grozinger C. M. (2014). Genomic analysis of the interaction between pesticide exposure and nutrition in honey bees (*Apis mellifera*). J. Insect Physiol. 71, 177–190. 10.1016/j.jinsphys.2014.10.002 25450567

[B129] Schmidhempel P. (1998). Parasites in social insects. Q. Rev. Biol. 12, 83–95.

[B130] Schmid-Hempel P. (2005). Evolutionary ecology of insect immune defenses. Annu. Rev. Entomol. 50, 529–551. 10.1146/annurev.ento.50.071803.130420 15471530

[B131] Schneider C. W. Tautz J. Grunewald B. Fuchs S. (2012). RFID tracking of sublethal effects of two neonicotinoid insecticides on the foraging behavior of *Apis mellifera*. PLoS ONE 7:e30023. 10.1371/journal.pone.0030023 22253863 PMC3256199

[B132] Simone M. Evans J. D. Spivak M. (2009). Resin collection and social immunity in honey bees. Evolution 63, 3016–3022. 10.1111/j.1558-5646.2009.00772.x 19619221

[B133] Soltanian S. Stuyven E. Cox E. Sorgeloos P. Bossier P. (2009). Beta-glucans as immunostimulant in vertebrates and invertebrates. Crit. Rev. Microbiol. 35, 109–138. 10.1080/10408410902753746 19514911

[B134] Southwick E. Heldmaier G. (1987). Temperature control in honey bee colonies. Bioscience 37, 395–399. 10.2307/1310562

[B135] Torto B. Suazo A. Alborn H. Tumlinson J. H. Teal P. E. A. (2005). Response of the small hive beetle (*Aethina tumida*) to a blend of chemicals identified from honeybee (*Apis mellifera*) volatiles. Apidologie 36, 523–532. 10.1051/apido:2005038

[B136] Trenczek T. (1998). Endogenous defense mechanisms of insects. Zoology 101, 298–315.

[B137] Tsvetkov N. Samson-Robert O. Sood K. Patel H. S. Malena D. A. Gajiwala P. H.. (2017). Chronic exposure to neonicotinoids reduces honey bee health near corn crops. Science 356, 1395–1397. 10.1126/science.aam7470 28663503

[B138] Underwood R. M. Currie R. W. (2003). The effects of temperature and dose of formic acid on treatment efficacy against *Varroa destructor* (Acari: Varroidae), a parasite of *Apis mellifera* (Hymenoptera: Apidae). Exp. Appl. Acarol. 29, 303–313. 10.1023/A:1025892906393 14635816

[B139] Vanbergen A. (2013). Threats to an ecosystem service: pressures on pollinators. Front. Ecol. Environ. 11, 251–259. 10.1890/120126

[B140] Vanengelsdorp D. Evans J. D. Saegerman C. Mullin C. Haubruge E. Nguyen B. K.. (2009). Colony collapse disorder: a descriptive study. PLoS ONE 4:e6481. 10.1371/journal.pone.0006481 19649264 PMC2715894

[B141] Vojvodic S. Jensen A. B. James R. R. Boomsma J. J. Eilenberg J. (2011). Temperature dependent virulence of obligate and facultative fungal pathogens of honeybee brood. Vet. Microbiol. 149, 200–205. 10.1016/j.vetmic.2010.10.001 21050682

[B142] Waller G. D. Carpenter E. W. Ziehl O. A. (1972). Potassium in onion nectar and its probable effect on attractiveness of onion flowers to honey bees. J. Econ. Entomol. 97, 535–539.

[B143] Wang K. Hu L. Jin X. L. Ma Q. X. Marcucci M. C. Netto A. A. L. (2015). Polyphenol-rich propolis extracts from China and brazil exert anti-inflammatory effects by modulating ubiquitination of traf6 during the activation of NF-κb. J. Funct. Foods 19, 464–478. 10.1016/j.jff.2015.09.009

[B144] Wang K. Jin X. L. Shen X. G. Sun L. P. Wu L. M. Wei J. Q.. (2016). Effects of Chinese propolis in protecting bovine mammary epithelial cells against mastitis pathogens-induced cell damage. Mediators Inflamm. 2016:8028291. 10.1155/2016/8028291 27433029 PMC4940570

[B145] Wang Y. Campbell J. B. Kaftanoglu O. Page R. E. Jr. Amdam G. V. Harrison J. F. (2016). Larval starvation improves metabolic response to adult starvation in honey bees (*Apis mellifera* L.). J. Exp. Biol. 219, 960–968. 10.1242/jeb.136374 27030776

[B146] Wang Y. Kaftanoglu O. Fondrk M. K. Page R. E. (2014). Nurse bee behaviour manipulates worker honeybee (*Apis mellifera* L.) reproductive development. Anim. Behav. 92, 253–261. 10.1016/j.anbehav.2014.02.012

[B147] Wenning C. J. (2001). Spread and threat of the small hive beetle. Am. Bee J. 141, 2857–2861.

[B148] Wilson M. B. Pawlus A. D. Brinkman D. Gardner G. Hegeman A. D. Spivak M.. (2017). 3-Acyl dihydroflavonols from poplar resins collected by honey bees are active against the bee pathogens *Paenibacillus larvae* and *Ascosphaera apis*. Phytochemistry 138, 83–92. 10.1016/j.phytochem.2017.02.020 28258722

[B149] Wolf S. McMahon D. P. Lim K. S. Pull C. D. Clark S. J. Paxton R. J.. (2014). So near and yet so far: harmonic radar reveals reduced homing ability of Nosema infected honeybees. PLoS ONE 9:e103989. 10.1371/journal.pone.0103989 25098331 PMC4123971

[B150] Woyciechowski M. Czekonska K. (2014). The effect of temperature on nosema apis zander (microsporida, nosematidae) infection in honey bees (*Apis mellifera*). Paras. J. De La Societe Francaise De Parasitol. 6, 185–187. 10.1051/parasite/1999062185

[B151] Wu Y. Dong X. Kadowaki T. (2017). Characterization of the copy number and Variants of Deformed Wing Virus (DWV) in the pairs of honey bee pupa and infesting varroa destructor or *Tropilaelaps mercedesae*. Front. Microbiol. 8:1558 10.3389/fmicb.2017.01558 28878743 PMC5572262

[B152] Yan H. Jia H. Gao H. Guo X. Xu B. (2013). Identification, genomic organization, and oxidative stress response of a sigma class glutathione S-transferase gene (AccGSTS1) in the honey bee, *Apis cerana cerana*. Cell Stress Chaperones. 18, 415–426. 10.1007/s12192-012-0394-7 23250585 PMC3682021

[B153] Yao P. Chen X. Yan Y. Liu F. Zhang Y. Guo X.. (2014). Glutaredoxin 1, glutaredoxin 2, thioredoxin 1, and thioredoxin peroxidase 3 play important roles in antioxidant defense in *Apis cerana cerana*. Free Radic. Biol. Med. 68, 335–346. 10.1016/j.freeradbiomed.2013.12.020 24389255

[B154] Yao P. Hao L. Wang F. Chen X. Yan Y. Guo X.. (2013). Molecular cloning, expression and antioxidant characterisation of a typical thioredoxin gene (AccTrx2) in *Apis cerana cerana*. Gene 527, 33–41. 10.1016/j.gene.2013.05.062 23747404

[B155] Yu F. Kang M. Meng F. Guo X. Xu B. (2011). Molecular cloning and characterization of a thioredoxin peroxidase gene from *Apis cerana cerana*. Insect Mol. Biol. 20, 367–378. 10.1111/j.1365-2583.2011.01071.x 21382109

[B156] Yue D. Nordhoff M. Wieler L. H. Genersch E. (2008). Fluorescence in situ hybridization (FISH) analysis of the interactions between honeybee larvae and *Paenibacillus larvae*, the causative agent of American foulbrood of honeybees (*Apis mellifera*). Environ. Microbiol. 10, 1612–1620. 10.1111/j.1462-2920.2008.01579.x 18331334

[B157] Zhang J. Zhang Y. Han R. (2016). The high-throughput production of dsRNA against sacbrood virus for use in the honey bee *Apis cerana* (Hymenoptera: Apidae). Virus Genes 52, 698–705. 10.1007/s11262-016-1346-6 27139728

[B158] Zhang X. He S. Y. Evans J. D. Pettis J. S. Yin G. F. Chen Y. P. (2012). New evidence that deformed wing virus and black queen cell virus are multi-host pathogens. J. Invertebr. Pathol. 109, 156–159. 10.1016/j.jip.2011.09.010 22001629

[B159] Zhang Y. Liu Y. Guo X. Li Y. Gao H. Guo X.. (2014). sHsp22.6, an intronless small heat shock protein gene, is involved in stress defence and development in *Apis cerana cerana*. Insect Biochem. Mol. Biol. 53, 1–12. 10.1016/j.ibmb.2014.06.007 25008786

[B160] Zhang Y. Yan H. Lu W. Li Y. Guo X. Xu B. (2013). A novel omega-class glutathione S-transferase gene in *Apis cerana cerana*: molecular characterisation of GSTO2 and its protective effects in oxidative stress. Cell Stress Chaperones 18, 503–516. 10.1007/s12192-013-0406-2 23382010 PMC3682018

[B161] Zhang Y. Y. Guo X. L. Liu Y. L. Liu F. Wang H. F. Guo X. Q.. (2016). Functional and mutational analyses of an omega-class glutathione S-transferase (GSTO2) that is required for reducing oxidative damage in *Apis cerana cerana*. Insect Mol. Biol. 25, 470–486. 10.1111/imb.12236 27170478

[B162] Zhao G. Wang C. Wang H. Gao L. Liu Z. Xu B.. (2018). Characterization of the CDK5 gene in *Apis cerana cerana* (AccCDK5) and a preliminary identification of its activator gene, AccCDK5r1. Cell Stress Chaperones 23, 13–28. 10.1007/s12192-017-0820-y 28674940 PMC5741578

[B163] Zheng H. Q. Gong H. R. Huang S. K. Sohr A. Hu F. L. Chen Y. P. (2015). Evidence of the synergistic interaction of honey bee pathogens *Nosema ceranae* and Deformed wing virus. Vet. Microbiol. 177, 1–6. 10.1016/j.vetmic.2015.02.003 25752367

